# Blockage of TGF-*α* Induced by Spherical Silica Nanoparticles Inhibits Epithelial-Mesenchymal Transition and Proliferation of Human Lung Epithelial Cells

**DOI:** 10.1155/2019/8231267

**Published:** 2019-02-18

**Authors:** YiXun Li, Huan Li, Yong Duan, XueMei Cai, DingYun You, Feng Zhou, ChuanQi Yang, XiaoYu Tuo, ZiJie Liu

**Affiliations:** ^1^Yunnan Key Laboratory of Laboratory Medicine, Kunming, Yunnan, China; ^2^Department of Laboratory Medicine, The First Affiliated Hospital of Kunming Medical University, Kunming, Yunnan, China; ^3^School of Public Health, Kunming Medical University, Kunming, Yunnan, China

## Abstract

*Background*. Xuanwei City in Yunnan province has been one of the towns with highest lung cancer mortality in China. The high content of amorphous silica in the bituminous coal from Xuanwei of Yunnan is mainly present as irregular and spherical silica nanoparticles (SiNPs). It has been reported that silica nanoparticles in bituminous coal correlated with the high incidence of lung cancer in Xuanwei. To explore the role and mechanism of SiNPs in the tumorigenesis of lung cancer in Xuanwei, human mononuclear cells (THP-1) and human bronchial epithelial cells (BEAS-2B) were cocultured in a transwell chamber. Combined with Benzo[*a*]pyrene-7, 8-dihydrodiol-9, and 10-epoxide (BPDE), SiNPs could significantly promote the proliferation and Epithelial-Mesenchymal Transition (EMT) and inhibit apoptosis of BEAS-2B cells and induce the release of TGF-*α* from THP-1 cells. After neutralizing TGF-*α* with antibody, the proliferation and EMT were decreased and enhanced apoptosis of BEAS-2B cells. Furthermore, the results showed that TGF-*α* in the sera of patients with lung adenocarcinoma in Xuanwei were significantly higher than in patients with benign pulmonary lesions in Xuanwei and those with lung adenocarcinoma in outside of Xuanwei of Yunnan. Taken together, our study found that SiNPs promoted the proliferation and EMT of BEAS-2B cells by inducing the release of TGF-*α* from THP-1 cells.

## 1. Introduction

Xuanwei City in Yunnan province has a high incidence of lung cancer, 20~30 times higher than in other regions of China [1]. The incidence of lung cancer in Xuanwei is related to the high content of silica nanoparticles in the local C1 coal seam of bituminous coal [2, 3]. The spatial distribution and mortality rate of lung cancer mortality coincide with the distribution of the C1 coal seam [3]. The silica nanoparticles in Xuanwei bituminous coal are amorphous, mainly irregular, and spherical (spherical silica nanoparticles, SiNPs) [4]. There is no definitive evidence that amorphous silica has a direct carcinogenic effect. Therefore, it is difficult to explain the phenomenon of high incidence of lung cancer in Xuanwei only by the theory of carcinogenesis of silica nanoparticles [5].

Lots of studies showed that crystalline silica nanoparticles have a carcinogenic effect [6–8]. It is believed that amorphous silica is relatively safe, and only one study reported the role of SiNPs in the tumorigenesis of lung cancer [9]. SiNPs are the smoothest amorphous silica. Increasing evidence shows that amorphous silica nanoparticles cause inflammation [10]. Thus, SiNPs in Xuanwei bituminous coal may also play an important role in the tumorigenesis of lung cancer by inducing inflammation. Inflammation induced by SiNPs may promote the tumorigenesis of lung cancer combined with the mutagenic effect of organic compounds found in the same coal, such as benzopyrene. The carcinogenic effect of benzopyrene* in vivo *is mainly achieved by its metabolites, Benzo[*a*]pyrene-7, 8-dihydrodiol-9, and 10-epoxide (BPDE). In this study, human Leukemic monocyte cell line (THP-1) and primary and immortalized human bronchial epithelial cells (BEAS-2B) cells were cocultured in a transwell chamber, primed with BPDE, and subsequently treated with SiNPs. Tumorigenicity assays were performed to determine the proliferation and Epithelial-Mesenchymal Transition (EMT)* in vitro *and* in vivo*. The cytokines in the supernatant of BEAS-2B and THP-1 cells were screened using a cytokine chip. A significant increase release of TGF-*α* was found in THP-1 cells after stimulation of SiNPs. Moreover, blockage of TGF-*α* pathway with antibody, the proliferation, and EMT were decreased and enhanced apoptosis of BEAS-2B cells* in vitro* and* in vivo*.

## 2. Materials and Methods

### 2.1. Ethical Standards

The study was approved by the Ethics Committee of Kunming Medical University, China (No. KMMC2017056). Animal work was performed in compliance with the guidelines established by the Kunming Medical University Institutional Animal Care and Use Committee (No. KMIACU2017043). All subjects signed informed consent.

### 2.2. Blood Samples

The blood samples after clinical examination were collected from the first and the third affiliated hospital of Kunming Medical University, From July 2017 to March 2018. 23 Xuanwei patients with lung adenocarcinoma were enrolled; those patients had lived in Xuanwei for more than 15 years and were diagnosed with lung adenocarcinoma by pathology and had received no systematic treatment. 25 patients outside of Xuanwei with lung adenocarcinoma were enrolled too; those come from Yunnan and have not lived in Xuanwei for more than 3 months and were diagnosed with lung adenocarcinoma by pathology and had received no systematic treatment. Meanwhile, 22 patients with benign pulmonary lesions in Xuanwei were detected, who had lived in Xuanwei for more than 15 years. Lung cancer was definitely excluded, and other pulmonary diseases were diagnosed, which include 13 cases of hamartoma, 6 cases of granuloma, and 3 cases of benign tumors. There were no bacterial and viral pulmonary infections. The sera of the patients were collected after clinical tests and stored in −80°C. Patient information was shown in Table 1.

### 2.3. Cell Lines and Cell Culture

BEAS-2B (KCB 200922YJ) and THP-1 (KCB 200549YJ) cells as well as LHC-9 medium were purchased from Kunming Institute of Zoology (Chinese Academy of Sciences, Kunming, China). BEAS-2B cells were cultured in LHC-9 medium, THP-1 cells were cultured in RPMI1640 medium (HyClone, USA) with 10% fetal Bovine serum (HyClone, USA), and BEAS-2B and THP-1 cells were supplemented with 1% penicillin and streptomycin (Thermo Fisher Scientific, USA). The cells were cultured at 37°C in a humidified atmosphere with 5% CO_2_.

### 2.4. Cell Viability

Cell Counting Kit-8 was to detect the viability of THP-1 and BEAS-2B cells. The cells were exposed to various concentrations of 0, 200, 400, 800, and 1000 nmol/L BPDE (MRIGlobal, Kansas, USA) or 0, 3.125, 6.25, 12.5, and 25 *μ*g/mL SiNPs (Aladdin, Shanghai, China) for 48 h. After replacing the RPMI 1640 medium, 10 *μ*L of CCK-8 reagent (Dojindo Molecular Technologies, Inc., Kumamoto, Japan) was added to each well, and the 96-well plate was incubated at 37°C for 1.5 h. The absorbance was measured at 450 nm by microplate reader (Thermo Fisher Scientific, USA). All the experiments were performed in triplicate.

### 2.5. Colony Formation Assay

BEAS-2B cells were seeded into the lower transwell chamber (3412, Costar, USA) and treated by 800 nmol/L BPDE solved in DMSO and incubation for 12 hours, THP-1 cells were seeded into the upper chamber, and 12.5*μ*g/mL SiNPs suspended in sterile PBS were added. The morphology of silica nanoparticles used in this study was analyzed by scanning electron microscope at the Advanced Analysis and Measurement Center of Yunnan University. The BEAS-2B cells were collected after 24 hours. In the cloning assay after neutralization with antibodies, an anti-TGF*α* antibody (1.6 ng/mL; ab9585, Abcam, USA) and TGF-*α* (0.4 ng/mL, 239-A, R&D systems, USA) were added, respectively. After digestion with 0.25% (v/v) of trypsin solution (Thermo Fisher Scientific, USA), the BEAS-2B cells were dispersed into a single-cell suspension and the cell density adjusted to 1 × 10^4^ cells/mL. The wells of a 6-well plate were filled with 1 mL of 0.4% agar and 100 *μ*L of single-cell suspension, mixed, and cultured on 0.8% agar for 14 days to observe the colony formation and calculate the colony formation rate (number of clones/number of cells in the inoculum × 100%). Each test was repeated three times, the cloning rate of each group was calculated, and the mean and standard deviation of the colony formation rate were calculated.

### 2.6. Mouse Xenograft

The BEAS-2B cells primed with BPDE (800 nmol/L) treated with or without 12.5 *μ*g/mL SiNPs were cultured for three generations and 0.2 mL BEAS-2B cells of a 1 × 10^6^ cells/mL suspension were inoculated subcutaneously into the flank of male BALB/c nude mice (Animal experiment center of Kunming Medical University, China). Six mice were inoculated in each group, and each mouse was inoculated with 2 sites. The measurement of tumor volume when the tumors were visible to the naked eye was conducted every 2 days thereafter. The tumor volume was calculated using the formula: V (mm^3^) = A (mm) × B^2^ (mm^2^) ×0.5 (A, length; B, width) [11]. The tumor growth curve was plotted with time as the transverse coordinate and the tumor volume as the ordinate. After 25 days, the mice were sacrificed, and the tumor tissues were measured and stored at −80°C.

### 2.7. Analysis of Cytokines

BEAS-2B cells were primed with 800 nmol/L BPDE for 12 hours, and THP-1 cells were starved in a reduction medium based on RPMI 1640 medium for 12 hours. The THP-1 cells were then resuspended in LHC-9 medium and added to the upper chamber, followed by cultivation with or without 12.5 *μ*g/mL SiNPS for 48 hours. The culture medium was collected and cleared by centrifugation at 5000×g for 10 min. The release of cytokines and chemokines was analyzed using a Human XL Proteome Profiler™ Array, in conjunction with the Cytokine Array Kit (cat No: ARY022B, R&D Systems, USA). Export signal values and the average signals (pixel density) of the pairs of duplicate spots were determined and compared to corresponding signals on different arrays to determine the relative change between the controls and treated by BPDE and SiNPs.

### 2.8. Immunohistochemistry (IHC)

The paraffin-embedded tissue sections were deparaffinized, dehydrated, and boiled for 15 min in 0.01 M phosphate buffer (PBS, pH 7.2). Endogenous peroxidases were blocked by incubation in 3% hydrogen peroxide for 30 min. Outside specific binding sites were blocked with 10% normal goat serum (Boster, Wuhan, China) for 30 min at 37°C, after which the tissue samples were incubated at 4°C overnight with the following primary antibodies: anti-E-cadherin (ab76055, Abcam, USA), anti-vimentin (ab92547, Abcam, USA), anti-pan cytokeratin (ab215838, Abcam, USA), and anti-fibronectin (30339, Promab, USA). Washed with PBS, then samples were incubated with secondary peroxidase-conjugated antibodies for 30 min at 37°C and were visualized with 2% 3, 3-diaminobenzidine tetrachloride (Maixin, China). Finally, the sections were counterstained with hematoxylin (Maixin, China). The negative control group went through the same steps as described above except for replacing the primary antibody with phosphate buffered saline (PBS). All slides were observed under a BX53 Light Microscope (Olympus, Japan).

### 2.9. Immunocytochemistry

The BEAS-2B cells cocultured with THP-1 cells pretreated with 800 nmol/L BPDE and 12.5 *μ*g/mL SiNPs were fixed on slides with 4% paraformaldehyde and air-dried. The subsequent steps were the same as described for immunohistochemistry.

### 2.10. Relative Quantitative Real-Time Polymerase Chain Reaction (qRT-PCR)

Total RNA was reverse transcribed using RevertAid™ H Minus First Strand cDNA Synthesis Kit (Fermentas, Thermo Fisher Scientific, USA). The mRNA levels were determined using a HT7900Real-Time PCR system (Applied Biosystems, USA) with SYBR Green PCR Master Mix (Applied Biosystems,, USA), and each sample was measured 3 times. Relative expression levels were calculated according to the comparative threshold cycle method (2^-ΔΔCt^) using GAPDH as an endogenous control. Primers for expression analysis were synthesized by Sangon Biotech (shanghai, China), as follows: TGF-*α*: F 5′-AGGTCCGAAAACACTGTGAGT-3′, R 3′-AGCAAGCGGTTCTTCCCTTC-5′; GAPDH: F 5′-GGAGCGAGATCCCTCCAA AAT-3′, R 3′-GGCTGTTGTCATACTTCTCATGG-5′.

### 2.11. Western Blot Analysis

Total protein was extracted by Total Protein Extraction Kit (ProMab, USA). The total protein concentration was measured using the Bio-Rad DC Protein Assay kit (Bio-Rad, Hercules, USA). An aliquot comprising 20 *μ*g of total protein was used for SDS-polyacrylamide gel electrophoresis (SDS-PAGE). The separated protein bands were transferred to a polyvinylidene difluoride (PVDF) membrane after blocking with defatted milk at 37°C for 2 hours and incubated at 4°C overnight with anti-PCNA antibody (2586, Cell Signaling Technology, USA) and ki67 antibody (BM438, Boster, China); antibodies for proteins involved in EMT used the same as IHC method, goat anti-mouse and rabbit (ab205719, ab205718) as second antibody. Immunoreactive proteins were detected using enhanced chemiluminescence (NCI4106, PIERCE, USA). Band was analyzed by Image-Pro Plus 6.0. Each experiment was performed in triplicate.

### 2.12. TUNEL Assay

The tumor tissue was sliced, dewaxed, and grilled for 4 hours. To detect apoptosis, tumor sections were assessed using a terminal deoxynucleotidyl transferase-mediated dUTP nicked end labeling (TUNEL) assay kit (Calbiochem TdT-FragEL™ DNA Fragmentation Detection Kit, Calbiochem, Germany), according to the manufacturer's instructions.

### 2.13. ELISA

Cell culture supernatants or patient sera were analyzed using the TGF-*α* ELISA kit purchased from Xinbosheng Biological Technology (Shenzhen, China) and operated according to the manufacturer's instructions. The OD_450_ value was measured in a Multiskan Sky microplate reader (Thermo Fisher Scientific, USA).

### 2.14. Neutralization of TGF-*α*

BEAS-2 cells were treated with 0.4 ng/mL rabbit anti-TGF-*α*antibody (ab9585, Abcam, USA) relative to IgG control (ab172730, Abcam, USA). Then, BEAS-2 and THP-1 cells were cocultured in transwell chambers and treated with 800 nmol/L BPDE and 12.5 *μ*g/mL SiNPs for 48 h. EMT markers were analyzed in xenograft tissues by western blotting.

### 2.15. Statistical Analysis

Statistical analysis was performed using SPSS software version 17.0 (IBM Corp., USA). Measurement data with a normal distribution were expressed as means ± SD. The data with a partial distribution are represented by the median (minimum, maximum). For variables with two groups, the *t*-test was used for parametric analysis and the Mann-Whitney test was used as a nonparametric analysis. Differences with* p*-values less than 0.05 were considered statistically significant.

## 3. Results

### 3.1. SiNPs Promotes the Proliferation and EMT of BEAS-2B Cells

**Figure 1:**
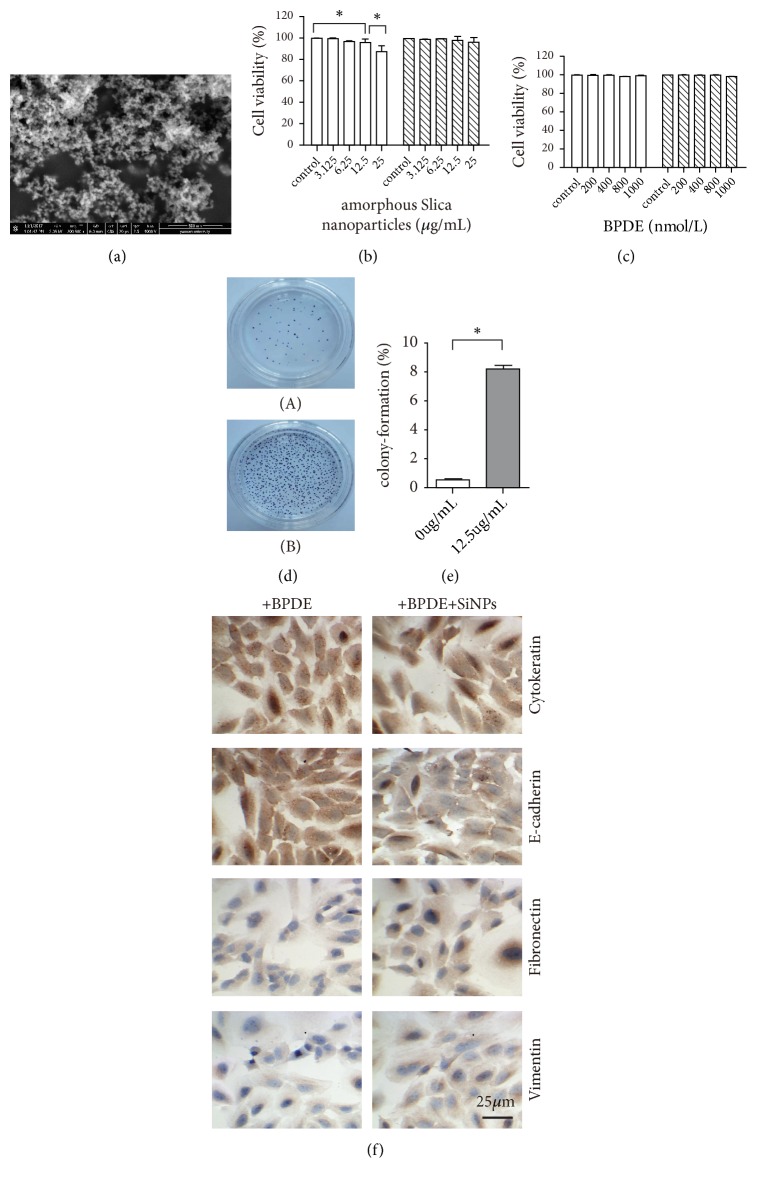
Effect of SiNPs on the clonogenicity and EMT of BEAS-2B cells. (a) SiNPs were analyzed by electron microscope. (b) Cell viability at different concentrations of SiNPs. (c) Cell viability at different concentrations of BPDE. (d) Soft agar clonogenicity was assayed used BEAS-2B cells were treatment of BPDE without SiNPs (A) or with SiNPs (B). (e) Compared with the BPDE treated alone, the number of clones increased significantly after treatment of SiNPs (12.5 *μ*g/mL). (f) Immunohistochemistry for EMT markers of BEAS-2B cells. *∗* p < 0.05.

The silica particles were spherical with a uniform size of about 30~50nm, with good dispersion and no obvious agglomeration (Figure 1(a)), and the optimum concentrations of SiNPs and BPDE were 12.5 *μ*g/mL and 800 nmol/L, respectively (Figures 1(b) and 1(c)). To explore whether SiNPs can promotes the proliferation of BEAS-2B, we used soft agar clonogenicity assays. The results showed that the colony formation rate of BEAS-2B cells treated with BPDE and SiNPs was 8.54%  ±  0.21%, which was significantly higher than that with treatment of BPDE alone (0.65%  ±  0.12%) (p<0.005) (Figures 1(d) and 1(e)). EMT endows lung cancer cells with the ability to metastasize and invade, including acquiring stem cell characteristics, reducing apoptosis and aging, and promoting immunosuppression. During the process of EMT in epithelial cells, many proteins are upregulated or downregulated to play an important role. These proteins can be used as markers of EMT in lung cancer, including epithelial markers cytokeratin and E-cadherin, and mesenchymal markers fibronectin and vimentin [12, 13].

The expression of cytokeratin and E-cadherin in the BEAS-2B cells treated with SiNPs was significantly downregulated, while the expression of fibronectin and vimentin was upregulated (Figure 1(f)). The results therefore demonstrated that the proliferating BEAS-2B cells had undergone EMT.

### 3.2. SiNPs Accelerates the Growth of Subcutaneous Xenograft

**Figure 2:**
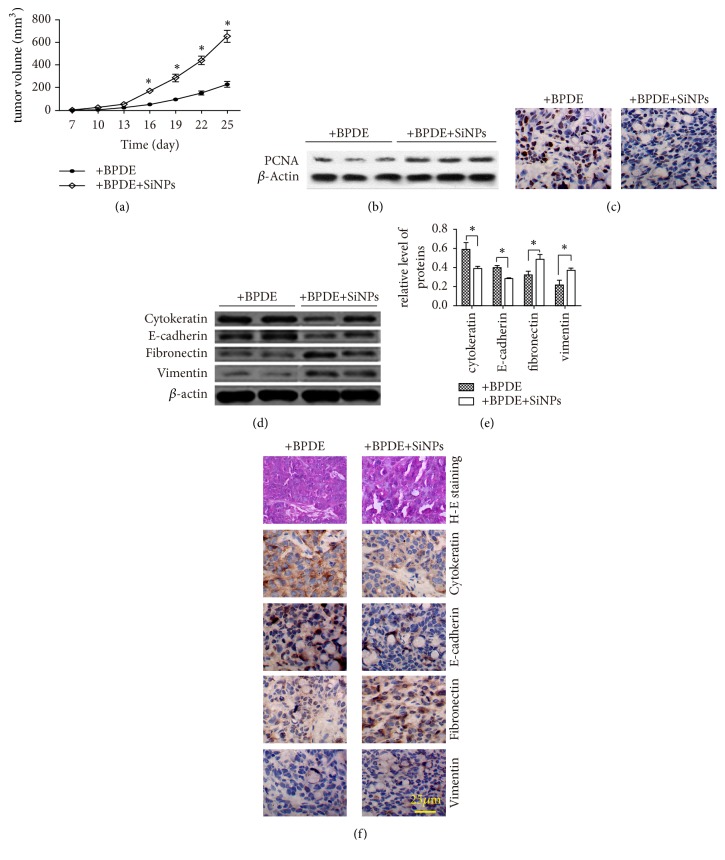
SiNPs promote EMT and tumor growth* in vivo*. (a) The growth curve of xenografted tumor. (b) PCNA was detected by western blotting in tumor tissues. (c) Apoptosis was analyzed by TUNEL assay in tumor tissues. (d, e, and f) proteins involved in EMT were detected by western blotting and immunohistochemistry in tumor tissues. *∗* p < 0.05.H-E staining: hematoxylin-eosin staining.

BEAS-2B cells were implanted into nude mice and the tumor tissues were removed after 25 days. The volume of xenograft tumors was significantly increased when the BEAS-2B cells were treated with BPDE and SiNPs comparing to that of cells treatment of BPDE alone (Figure 2(a)). Proliferating cell nuclear antigen (PCNA) was also highly expressed in tissues which cells treated by BPDE and SiNPs, consistent with its role in cell proliferation and DNA synthesis (Figure 2(b)), and the apoptosis was decreased (Figure 2(c)). Subsequently, expression of cytokeratin and E-cadherin was detected. The results showed a decrease of cytokeratin and E-cadherin expression, while fibronectin and vimentin expressed higher compare with control (Figures 2(d), 2(e), and 2(f)). These results indicated that SiNPs could promote cell proliferation and EMT, inhibit the apoptosis of BEAS-2B cells, and promote tumor growth.

### 3.3. SiNPs Combined with BPDE Stimulates the Secretion of TGF-*α* in THP-1 Cells

**Figure 3:**
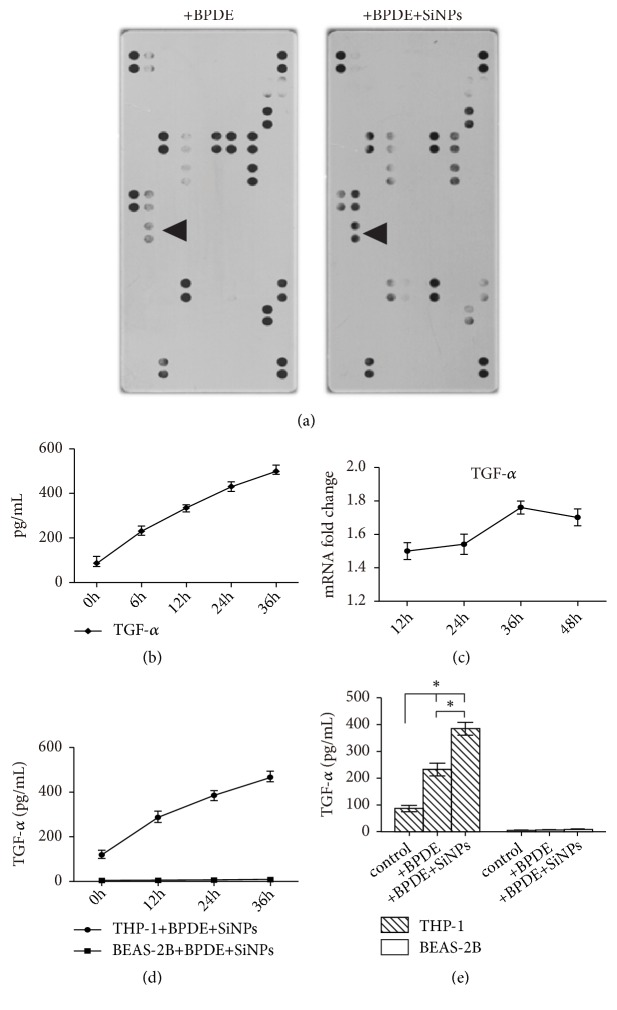
The expression of cytokines in the THP-1 and/or BEAS-2B. (a) Cytokines detected using a cytokine chip, arrows indicate the TGF-*α*. (b) TGF-*α* detected by ELISA in the culture medium of THP-1 cells treated with BPDE and SiNPs. (c) TGF-*α* mRNA detected by real-time PCR in THP-1 cells treated by SiNPs and BPDE. (d) TGF-*α* was detected by ELISA in THP-1 or BEAS-2B cells treated by BPDE combined with SiNPs. (e) TGF-*α* detected in THP-1 or BEAS-2B cells treated by BPDE with or without SiNPs by ELISA. *∗ p*<0.005.

To explore the mechanism of SiNPs on EMT and proliferation of BEAS-2B cells, cytokine chip was employed. The cytokine chip results demonstrated that expression of TGF-*α* increased significantly in the cocultured supernatant of BEAS-2B and THP-1 cells after the treatment of SiNPs (Figure 3(a)). Moreover, the results of the ELISA were consistent with the results of the cytokine chip assay; the concentration of TGF-*α* in the supernatant of THP-1 cells increased continuously in 36 hours (Figure 3(b)). The expression levels of TGF-*α* mRNA were increased when cells had treatment of SiNPs after 24 hours (Figure 3(c)).

To determine whether TGF-*α* was secreted by THP-1 cells or not, TGF-*α* in BEAS-2B and THP-1 cells supernatant treated with BPDE and SiNPs was detected by ELISA. The result showed that TGF-*α* in the supernatants of THP-1 cells increased continually in 36 hours but not in that of BEAS-2B cells (Figure 3(d)). These results indicated that TGF-*α* was mainly secreted by the THP-1 cells in the coculture system. At the same time, in order to study the effect of SiNPs on TGF-*α* secretion, we detected TGF-*α* in supernatants of BEAS-2B or THP-1 cells treated by BPDE with or without SiNPs. It was found that BPDE could induce of TGF-*α* in THP-1 cells, and TGF-*α* increased significantly after SiNP treatment, which showed that SiNPs could significantly stimulate the secretion of TGF-*α* (Figure 3(e)).

### 3.4. Blockage of TGF-*α* with Antibody Inhibit Cell Proliferation, EMT, and the Growth of Xenograft

**Figure 4:**
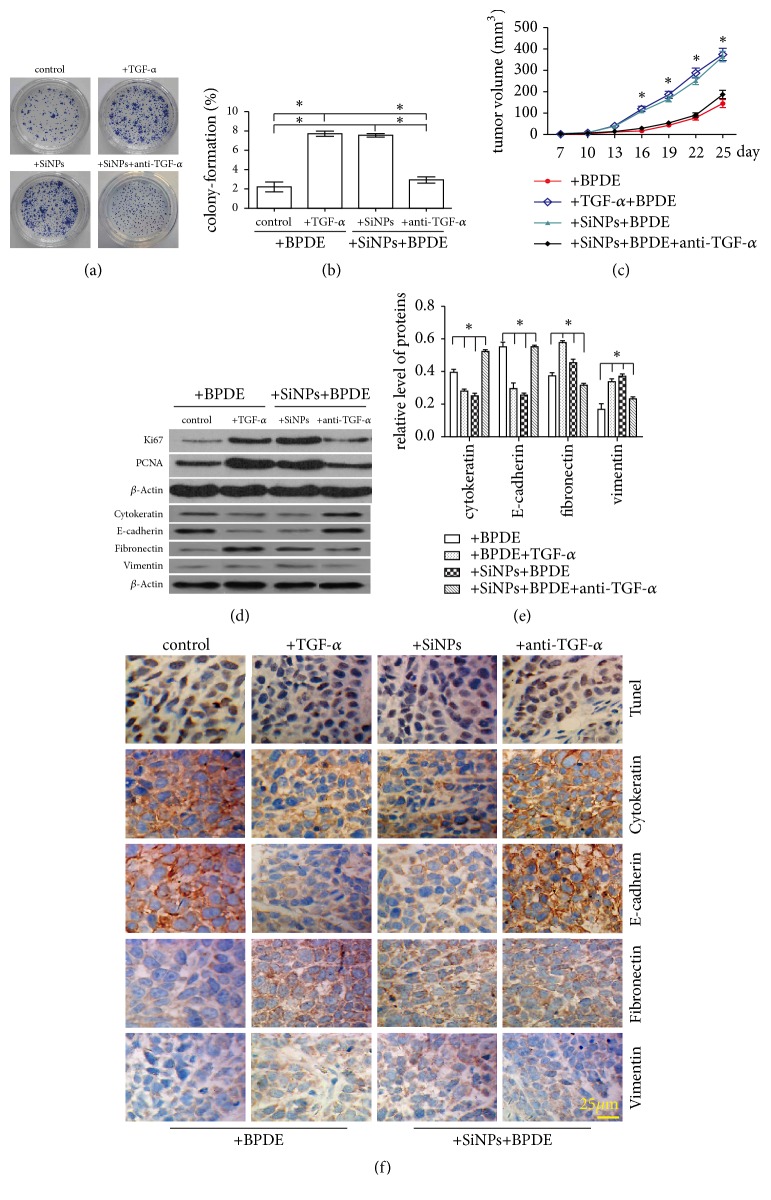
Effects of TGF-*α* and its neutralizing antibodies on the EMT of BEAS-2B cells and tumor growth. (a) The effects of TGF-*α* (0.4 ng/mL) and anti-TGF-*α* (1.6 ng/mL) on the proliferation of BEAS-2B cells, control group treated only by BPDE. (b) The colony formation rate of BEAS-2B cells. (c) The growth rate curve of xenografted tumors. (d, e) Ki67, PCNA, and proteins involved EMT in tumor tissue were detected by western blotting. (f) The apoptosis of the tumor tissue was detected using the TUNEL assay, and the markers of EMT in tumor tissue were detected by immunohistochemistry in tumor tissues.

Our results have been demonstrated that SiNPs stimulated a secretion of TGF-*α* in THP-1 cells. TGF-*α* is a transforming growth factor that is a ligand for the epidermal growth factor receptor, which activates a signaling pathway for cell proliferation, differentiation, and development. It has been associated with many types of cancers [14]. Subsequently, a neutralized antibody of TGF-*α* was employed to block TGF-*α*. The results showed that compared with group treated by TGF-*α* or SiNPs, colony formation rate and the proliferation were decreased while neutralized of TGF-*α* (Figures 4(a) and 4(b)). The volume of tumors was significantly reduced, and the growth rate of tumor was slowed (Figure 4(c)). The expression of Ki67 and PCNA was decreased (Figure 4(d)), while apoptosis was inhibited (Figure 4(f), top panel). Moreover, compared to tumor tissues treated by TGF-*α* or SiNPs, the expression of cytokeratin and E-cadherin was increased, and the expression of fibronectin and vimentin was decreased in treatment of TGF-*α* antibody (Figures 4(d), 4(e), and 4(f)); while being treated with rat IgG, there was no significant change of EMT, proliferation, and apoptosis compared with treated by TGF-*α* or SiNPs (data not shown).

### 3.5. TGF-*α* in the Sera of the Patients with Lung Adenocarcinoma in Xuanwei Is Higher than That of the Patients in Outside of Xuanwei Area and Xuanwei Patients with Benign Pulmonary Lesions

TGF-*α* in lung adenocarcinoma patients in Xuanwei (median, 20.3 pg/mL) was higher than that of patients with benign lung lesions (median, 1.8 pg/mL,* p*<0.005) and lung adenocarcinoma in outside of Xuanwei of Yunnan when patient less than 60 (median, 5.9 pg/mL in <50 years and 8.5 pg/mL in 50<years⩽60, and* p*<0.005).

## 4. Discussion

Amorphous silica is considered to be relatively safe, and there is no consensus that amorphous silica has direct carcinogenic effects. However, our results suggested that SiNPs combined with BPDE could promote EMT, proliferation of BEAS-2B cells and tumor growth.

Although amorphous silica got cytotoxicity, there is still a lack of evidence of its carcinogenicity, and only one in vivo study found that the incidence of lung cancer was increased by amorphous silica nanoparticles [9]. In vitro studies have shown that amorphous silica nanoparticles cannot cause mutations, but they can accelerate the malignant transformation of cells in the presence of mutations [15]. The results were in agreement with this study, and we found that SiNPs can enhance EMT of BEAS-2B cells combined with BPDE.

Do SiNPs enhance EMT and proliferation of BEAS-2B cells through inflammation? At present, it is considered that chronic inflammation is related to the tumorigenesis and development of cancer. However, rats only showed an acute inflammatory response within 8 hours after inhaling amorphous silica nanoparticles for a short time [16]. Even after long-term inhalation, most animals only showed emphysema and hyperplasia of alveolar cells [17]. In this study, we found that the secretion of TGF-*α* in THP-1 cells increased continuously after treatment with SiNPs and BPDE within 36 h. This result was somewhat different from the reported animal studies. This may be due to the removal of inhaled silica nanoparticles from the respiratory tract by animals, so that the duration of inflammation is relatively short.* In vitro*, SiNPs have act on BEAS-2B cells continuously, inducing a continuous inflammatory response. Another alternative reason is that SiNPs are more difficult to remove through the respiratory tract than other types of amorphous silica or crystalline silica due to their smooth surface, which may cause chronic inflammatory. However, it requires more evidences to support in future researches.

It was reported that amorphous silica nanoparticles can induce the production of IL-1*β*, IL-6, IL-8, TNF-*α*, MCP-1and other cytokines after acting on different cell types [18–20]. TGF-*β* play a very important role in tumorigenesis and development in cancer, many studies pay extraordinary attention on TGF-*β* [21, 22]. Skuland and Ovrevik found that amorphous silica nanoparticles can induce TGF-*α* release and which affect IL-6 and CXCL8 responses [20, 23]. In this case, we reported a release of TGF-*α* from THP-1 cells induced by SiNPs, which may be due to the size, concentration and surface characteristics of the nanoparticles [24–26]. Therefore, SiNPs may elicit different cytokine profiles. TGF-*α* is highly expressed in a variety of tumor tissues, and it can promote the growth and malignant transformation of ovarian cancer cells and prostate cancer cells [27, 28]. Moreover, it is also conducive to the invasion of other tumors such as liver cancer [29, 30]. Studies have shown that TGF-*α* is associated with the risk of lung cancer [31]. Our results showed that TGF-*α* could promote the proliferation and EMT of BEAS-2B cells, while the treatment with TGF-*α*-neutralizing antibodies significantly inhibited the proliferation and EMT of BEAS-2B cells. Furthermore, we found that TGF-*α* in the sera of the patients with lung adenocarcinoma in Xuanwei was higher than that of patients in outside Xuanwei areas of Yunnan or in Xuanwei patients with benign lung lesions. Although direct carcinogenesis of SiNPs has not been established to date, they can also promote the development of tumor combined with organic carcinogens, such as BPDE. This study provides a theoretical basis for explaining the high incidence of lung cancer in the Xuanwei district of Yunnan, China.

## 5. Conclusions

Our study found that SiNPs could promote the proliferation and EMT and inhibit the apoptosis of BEAS-2B cells to promote tumor growth by induced secretion of TGF-*α* in THP-1 cells combined with BPDE. Meanwhile, TGF-*α* in the sera of the patients with lung adenocarcinoma in Xuanwei was higher than in patients from outside of Xuanwei areas of Yunnan when patient less than 60 years or in Xuanwei patients with benign lung lesions. The results indicated that SiNPs could play an indirect role in the tumorigenesis and development of lung cancer by inflammation. This study provides a theoretical basis for explaining the high incidence of lung cancer in Xuanwei.

## Figures and Tables

**Table 1:** Characteristics of the study population (n=70).

	Lung adenocarcinoma in Xuanwei (n=23)	Lung adenocarcinoma in outside Xuanwei areas of Yunnan (n=25)	Benign pulmonary lesions in Xuanwei area (n=22)	*p* -value
Gender				
Male	10	12	12	0.757
Female	13	13	10	
Age (years)				
<50	9	5	12	0.001
>50, ≤60	12	5	6	
>60	2	15	4	
Smoking status				
Former/current	7	8	10	0.512
Never	16	17	12	
Stage of TNM				
IA, IB	14	10	/	0.272^$^
IIA, IIB	4	9	/	
IIIA, IV	5	6	/	

^$:^ Xuanwei lung cancer group compared with outside Xuanwei lung cancer group.

## Data Availability

The data used to support the findings of this study are available from the corresponding author upon request.
